# Evidence-Based Clinical Review on Cardiovascular Benefits of SGLT2 (Sodium-Glucose Co-Transporter Type 2) Inhibitors in Type 2 Diabetes Mellitus

**DOI:** 10.7759/cureus.9655

**Published:** 2020-08-11

**Authors:** Saad U Rehman, Faiqa Rahman

**Affiliations:** 1 Family Medicine, Primary Health Care Corporation, Doha, QAT

**Keywords:** sglt2 inhibitors, type 2 diabetes mellitus, cardiovascular effect, mortality, risk of cardiovascular diseases

## Abstract

Cardiovascular (CV) diseases are the leading cause of mortality and morbidity in patients with type 2 diabetes mellitus (DM). Therefore, there has been an increasing endorsement from diabetic associations across the globe for the use of anti-diabetic drugs, which not only provide not only glycemic control but also have cardioprotective effects. Sodium-glucose co-transporter type 2 (SGLT2) inhibitors are one class of drugs that have shown evidence of CV benefits in patients with type 2 DM. We reviewed the published literature and found five adequately powered clinical trials that evaluated the CV effects of SGLT2 inhibitors in type 2 DM patients. These trials assessed the CV effect of three SGLT2 inhibitors, namely, empagliflozin, canagliflozin, and dapagliflozin. It was found that all these clinical trials were multi-centric and conducted in and after 2015 across different parts of the World, enrolling type 2 DM patients with varied baseline characteristics in terms of age, BMI, sex, glomerular filtration rate, history of existing renal diseases, etc. In spite of these differences, the SGLT2 drugs were found to be beneficial by significantly reducing all-cause mortality, mortality due to CV causes, and risk of major CV events. All the studies highlighted the cardioprotective effect of SGLT-2 inhibitors, especially empagliflozin, dapagliflozin, and canagliflozin in type 2 DM patients with established CV disease, but the studies could not find significant improvement in 3P-MACE (three-point major adverse CV event) indicators offered by these drugs except empagliflozin. Hence, adequately powered clinical trials with long follow-up durations are the need of the hour to address this issue specifically.

## Introduction and background

The prevalence of diabetes mellitus (DM) has been estimated to be 463 million worldwide (9.3% of the total population). This burden is expected to increase to 578 million (10.2%) by 2030 and to 700 million (10.9%) by 2045 [1]. Four million deaths were attributed to diabetes in 2017, which posed a global health expenditure of USD 727 billion [2]. Out of total diabetic patients, 90% have type 2 DM (DM) [2]. Moreover, more than 70% of type 2 diabetes patients die of cardiovascular (CV) complications. The risk of mortality due to CV events is three times higher among diabetic patients as compared to age-matched patients without diabetes [3]. Therefore, it is imperative that anti-diabetic drugs developed so far should not only provide glycemic control but also has a cardioprotective mechanism to prevent CV events.

Before 2008, anti-diabetic agents focused on providing glycemic control. Nevertheless, due to better surveillance systems, especially for non-communicable diseases, and improved Civil Registration and Vital Statistics Systems worldwide in recent years, the risk of increased CV incidents among people with diabetes came to light [4]. It was found that some hypoglycemic drugs effectively lowered the blood glucose levels but paradoxically raised the adverse CV effect profile among the patients. For instance, the use of thiazolidinediones (rosiglitazone and pioglitazone) increased the risk of heart failure and myocardial infarction considerably among patients [5].

This prompted the U.S. Food and Drug Administration (FDA) and European Medicines Agency (EMA) to issue guidelines to the drug developers to investigate and rule out potential harmful CV effects of all new anti-diabetic agents after 2012 [6]. Therefore, it was decided to consider three-point major adverse CV event (3P-MACE) such as CV death, non-fatal stroke, or non-fatal myocardial infarction in the prospective CV outcome trials (CVOTs) for anti-diabetic drugs [7]. After this, there has been a steady stream of trials among diabetic patients with pre-existing CV disease (CVD), as this group of patients is more prone to CV related morbidity and mortality. Most of these trials have reported unprecedented CV benefit in secondary prevention of CVD and outcomes [8-11].

Sodium-glucose co-transporter type 2 inhibitors (SGLT2i) were introduced in 2013 as oral anti-diabetic drugs and have shown the possibility of being cardioprotective by demonstrating relative risk (RR) reduction of 3P-MACE [12]. In the first CVOT called Empagliflozin Cardiovascular Outcome Event Trial in Type 2 Diabetes Mellitus Patients (EMPA-REG OUTCOME), SGLT2 inhibitors showed promising CV benefits [13]. These drugs work by lowering blood glucose levels by reducing glucose renal reabsorption at proximal tubules in the kidney and thus excreting glucose in the urine. The mode of action of SGLT2i is independent of insulin and unlike any other anti-diabetic agents [13]. They are currently recommended as second-line therapy following metformin failure or intolerance [9,14]. Similar to the EMPA-REG-OUTCOME trial, various other clinical trials have been carried out among different populations and by administering different types of SGLT2i and have reported cardioprotective effects of these anti-diabetic drugs [15-17]. Moreover, the first renal outcome trial on SGLT2i has reported positive renal and CV benefits as well [18,19].

As there is limited existing evidence to compare various SGLT2i drugs directly, we planned to carry this review to summarize the clinical effect of different SGLT2i. Through this review, we aim to empower community health physicians in making informed decisions for choosing these drugs and prescribing an SGLT2i that best suits their patient depending on the duration of existing DM, absence or presence of any CV history, heart failure, renal failure, and any other co-morbidity.

## Review

Methods

Search Strategy

A search of scientific literature was conducted by two investigators S.R. and F.R. in PubMed, Embase, and Cochrane Central Register of Controlled Trials database 2013 onward. The search strategy used a combination of following keywords: “Randomized Controlled Trials”, “RCT”, “Type 2 Diabetes Mellitus”, “T2DM”, “sodium-glucose cotransporter-2 inhibitors”, “SGLT-2i”, “Canagliflozin”, “Dapagliflozin”, “Empagliflozin”, AND “major adverse cardiovascular events”, “mace”, “cardiovascular disease”, “coronary artery disease”, “coronary heart disease”, “myocardial infarction”, “cerebrovascular disease”, “mortality”, and “safety”. The keywords were checked for controlled vocabulary under Medical Subject Headings (MeSH) of PubMed.

Inclusion Criteria

We included all the randomized, placebo-controlled studies studying the effect of uptake of SGLT2i on CV outcomes such as CV death, myocardial infarction, and MACE on patients with T2DM and having a sample size of more than 2,000. All the full-text studies published in the English language until May 1, 2020, were included in the review.

Study Selection

**Figure 1:**
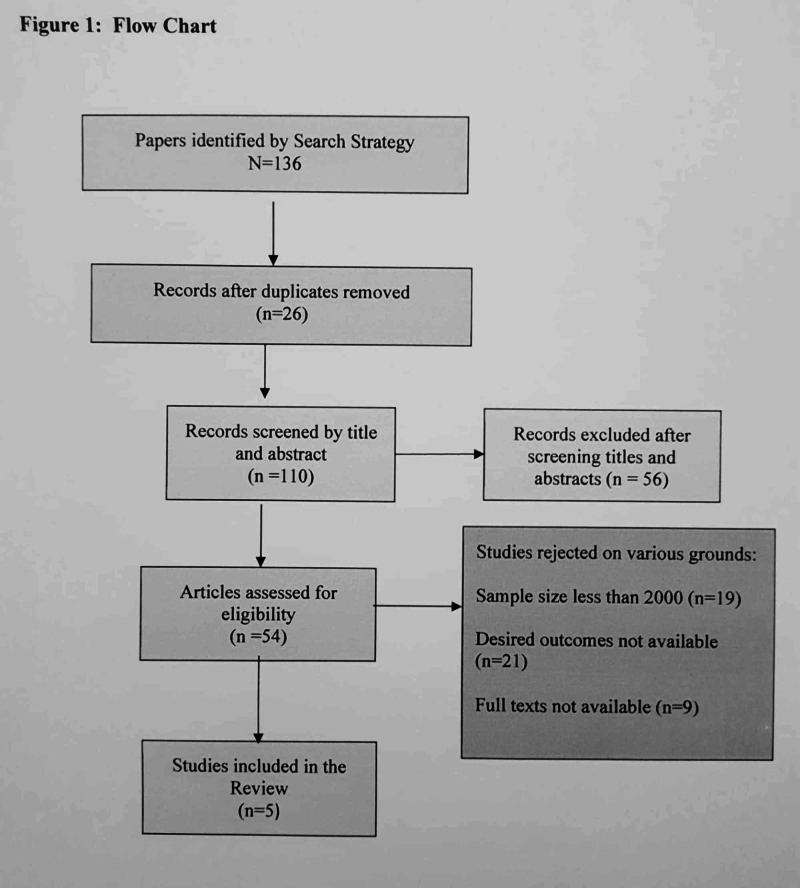
Flow chart of the study

Out of the total 136 studies identified through the literature search across various databases, 54 full-text studies were assessed after removing the duplicate studies and screening of the titles and abstracts. Finally, only five clinical trials were included, namely, EMPA-REG OUTCOME [13], Canagliflozin Cardiovascular Assessment Study (CANVAS) [7], Dapagliflozin Effect on Cardiovascular Events (DECLARE-TIMI 58) [12], Canagliflozin and Renal Endpoints in Diabetes with Established Nephropathy Clinical Evaluation (CREDENCE Trial) [9], and Dapagliflozin in Patients with Heart Failure and Reduced Ejection Fraction (DAPA-HF trial) [20] (Figure 1).

Data Extraction

Two independent authors assessed the eligibility of the identified studies. Any discrepancy in the inclusion of studies was discussed and sorted. A customized data extracted form was developed to extract the general and methodological data from the selected studies. The data includes the title of the study, authors, year of publication, study design, study area, study duration, sample size, participants, details of the intervention, comparison group, and outcome indicators.

Results

Characteristics of Included Trials

All of the aforementioned trials have been published after the year 2015, that is, EMPA-REG OUTCOME trial was published in September 2015 [13] and the DAPA-HF trial was published in the year September 2019 [20]. These trials were multi-centric multinational trials. All of these trials were phase 3 randomized controlled trials (RCTs). The total number of participants enrolled in trials varied to mention all trial patients numbers, which is from 4,401 in CREDENCE trial [9] to 17,160 in DECLARE-TIMI 58 [12]. The ratio between the intervention and comparison arm was 1:1 in all the trials.

The participants in all the trials had type 2 DM. However, the inclusion criteria in all the five trials were different. The age of included study varied from 18 years (in EMPA-REG and DAPA-HF) to more than 40 years in DECLARE-TIMI 58. The study population had a differential proportion of enrolled cases with established CVD; for instance, 99% of patients enrolled in EMPA, 66% in CANVAS, and 41% in DECLARE-TIMI 58 had established CVD. While the CREDENCE trial had enrolled patients with chronic kidney disease and was required to be on ACE/ARB (angiotensin-converting enzyme inhibitor/angiotensin receptor blocker) inhibitor therapy, the DAPA-HF trial enrolled patients with diagnosed heart failure on established therapy and a medical device. Also, the glomerular filtration rate, median exposure to trial, and premature discontinuation were different in the trials. The details of the trials are mentioned in Table 1.

**Table 1:** Characteristics of included trials ACE, angiotensin-converting enzyme inhibitors; ARB, angiotensin receptor blockers; BMI, body mass index; C, control group; CANVAS, Canagliflozin Cardiovascular Assessment Study; CV, cardiovascular; CREDENCE, Canagliflozin and Renal Endpoints in Diabetes with Established Nephropathy Clinical Evaluation; DAPA-HF, Dapagliflozin in Patients with Heart Failure and Reduced Ejection Fraction; CVD, cardiovascular diseases; CI, confidence interval; DECLARE-TIMI 58, Dapagliflozin Effect on Cardiovascular Events; eGFR, estimated glomerular filtration rate; EMPA-REG, Empagliflozin Cardiovascular Outcome Event Trial in Type 2 Diabetes Mellitus Patients; HbA1c, glycated hemoglobin (A1c); HR, hazard ratio; I, intervention group; NYHA, New York Heart Association functional classification; proBNP, natriuretic peptide tests measure levels; T2DM, type 2 diabetes mellitus; 3P-MACE, three-point composite of major adverse cardiovascular event: cardiovascular death, non-fatal myocardial infarction, and non-fatal stroke

Name of Trial and Publication Year	Countries and Median Follow-Up Time	Population	Number of Participants	Intervention Drug	Comparison	CV Outcome
EMPA-REG (September 2015) [13]	42 countries, 590 sites (3.1 years)	Age ≤ 18 years, T2DM, BMI = 45 or less, eGFR at least 30 ml/min/1.73 m^2^, established CVD, or any of two below: No intake of glucose-lowering agents for 12 weeks before randomization, HbA1c 7 to 9 Who had glucose-lowering agent 12 weeks before randomization and HbA1c of at least 7 and no more than 10	7020 (I=4687, C=2333)	Empagliflozin 10 mg or 25 mg	Standard care with placebo	Reduced 3P-MACE mortality among empagliflozin (10.5% vs. 12.1%; HR: 0.86; 95% CI: 0.74 - 0.99; p<0.001 for non-inferiority and p=0.04 for superiority). Significant reduction in all-cause mortality (5.7% vs 8.3%; HR: 0.68; 95% CI: 0.57-0.82; p≤0.001). Significant reduction in cardiovascular mortality (3.7% vs 5.9; HR: 0.62; 95% CI: 0.49-0.77; p≤0.001). Significant reduction in hospitalization for heart failure (2.7% vs 4.1%; HR: 0.65; 95% CI: 0.50-0.85; p≤0.002). Significant reduction in hospitalization for heart failure and death from cardiovascular causes excluding stroke (5.7% vs 8.5%; HR: 0.66; 95% CI: 0:55-0.79; p≤0.001).
CANVAS Program (CANVAS and CANVAS R) (June 2017) [7]	30 countries, 667 centers (2.4 years)	Age ≥ 30 years, with HbA1c ≥ 7 to 10.5 and history of symptomatic atherosclerotic CVD, age 50 years or more with two or more risk factors with CV (SBP>140 mg Hg, eGFR>30 ml/min/1.73 m^2^)	10142 (I=5795 C=4347)	Two groups, canagliflozin 100 mg and 300 mg daily	Placebo	Reduced 3P-MACE mortality in the intervention group (26.9 vs 31.5 participants with an event per 1000 patient-years; HR: 0.86; 95% CI: 0.75-0.97; p<0.001 for non-inferiority and p=0.02 for superiority).
DECLARE-TIMI 58(November 2018) [12]	882 sites in 33 countries (4.2 years)	Age ≥ 40 years, Hb1Ac from 6.5 to less than 12, creatinine clearance ≥ 60 mL/min), majority patients has no previous atherosclerotic CVD	17160 (I=8582, C=8578)	Dapagliflozin 10 mg	Placebo	No significant difference in the 3P-MACE in the DAPA group (8.8% vs 9.4%; HR: 0.93; 95% CI: 0.84-1.03; p=0.17). Significant reduction in cardiovascular death and hospitalization for heart failure (4.9% vs 5.8%; HR: 0.98; 95% CI: 0.83-0.95; p=0.005).
CREDENCE Trial (2019) [9]	34 countries (2.6 years)	Age ≥ 30 years, Hb1Ac 6.5 to 12, chronic kidney disease with eGFR of 30-90 ml/min/1.73 m^2^ with albuminuria (urine albumin creatinine ratio of >300 to 5000 mg/gm), on standard ACE or ARB therapy	4401 (I=2202, C=2199)	Canagliflozin 100 mg daily	Placebo	Significant reduction in 3P-MACE (9.9% vs 12.2%; HR: 0.80 95% CI: 0.67-0.95; p=0.01) Reduction in cardiovascular death (5.0% vs 6.4%; HR: 0.78; 95% CI: 0.61-1.00; p=0.05) Significant reduction in cardiovascular death and hospitalization for heart failure (8.1% vs 11.5%; HR: 0.69; 95% CI: 0.57-0.83; p≤0.001). Significant reduction in hospitalization for heart failure (4.0% vs 6.4%; HR: 0.61; 95% CI: 0.47–0.80; p≤0.001).
DAPA-HF Trial (September 2019) [20]	20 countries, 410 centers (18.2 months)	Age > 18 years, ejection fraction of 40% or less, NYHA class II, III, IV, proBNP 600 pg per mL, patients on the standard cardiovascular device and standard drug therapy	4744 (I=2373, C=2371)	Dapagliflozin 10 mg	Placebo	Significant reduction in hospitalizations for heart failure (9.7% vs 13.4%; HR: 0.70; 95% CI: 0.59-0.83). Significant difference in cardiovascular death (9.6% vs 11.5%; HR: 0.82; 95% CI: 0.69-0.98). Significant reduction in the all-cause mortality (11.6% vs 13.9%; HR: 0.83; 95% CI: 0.71-0.97). Significant reduction in hospitalization for heart failure or death from CVD (16.3% vs 21.2%; HR: 0.74; 95% CI: 0.65-0.85; p≤0.001).

Cardiovascular Outcomes

Table 1 represents the summary of all the trials included in this review, and the data presented are mainly a summary of the relevant CV outcomes shown as the composite of major adverse CV events, CV death, non-fatal myocardial infarction, non-fatal stroke (3P-MACE), hospitalization due to heart failure, and all-cause mortality.

The EMPA-REG OUTCOME trial had 7,020 patients with T2DM and coronary, peripheral, or cerebrovascular disease. They were randomized to receive two different doses of empagliflozin (10 or 25 mg) or placebo. The study reported that participants who received empagliflozin showed a significantly lower rate of the primary composite CV outcome (3P-MACE) and death from all causes of mortality compared to placebo [13].

The CANVAS Program comprised of two sister trials, the CANVAS and the CANVAS-Renal, which were designed to evaluate the CV safety and efficacy of canagliflozin in a person with diabetes with established CVD. This trial also showed a reduction in the 3P-MACE mortality but no significant difference in the reduction of all-cause mortality between the two groups [7].

In the DECLARE-TIMI 58 trial, a total of 17,160 patients with T2DM and established atherosclerotic CVD (ASCVD) or with multiple risk factors for ASCVD were randomized to receive either dapagliflozin 10 mg or placebo for a median period of 4.2 years [12]. This trial did not show any significant difference in 3P-MACE indicators. However, it demonstrated significantly reduced mortality due to CV causes and reduced rates of hospitalization due to heart failure [12].

All these trials did not individually enroll heart failure patients, but all of them demonstrated that SGLT2i is beneficial in patients with established CVD or patients with CV risk factors.

The CREDENCE trial presented its results following the above major studies. It enrolled people with diabetes with chronic kidney disease. The trial data were presented as primary renal outcomes and secondary CV outcomes for patients on canagliflozin compared to placebo. It showed a slight reduction of 3P-MACE but no difference in myocardial infarction and stroke [9].

In the DAPA-HF trial, for the first time, type 2 diabetics who had heart failure and patients with reduced ejection fraction were assigned to receive either dapagliflozin or placebo; in addition, patients were required to receive standard heart failure device and standard drug therapy. The trial showed a significant reduction in hospitalization for heart failure cases (described as an event for hospitalization or an urgent visit resulting in intravenous therapy for heart failure) and in CV death and all-cause mortality compared to the intervention group [20] (Table 2).

**Table 2:** Cardiovascular outcomes of the included trials CANVAS, Canagliflozin Cardiovascular Assessment Study; CREDENCE, Canagliflozin and Renal Endpoints in Diabetes with Established Nephropathy Clinical Evaluation; CV, cardiovascular; DAPA-HF, Dapagliflozin in Patients with Heart Failure and Reduced Ejection Fraction; DECLARE-TIMI 58, Dapagliflozin Effect on Cardiovascular Events; EMPA-REG, Empagliflozin Cardiovascular Outcome Event Trial in Type 2 Diabetes Mellitus Patients; MI, myocardial infarction; 3P-MACE: three-point composite of major adverse cardiovascular event (cardiovascular death, non-fatal myocardial infarction, and non-fatal stroke)

CVOTs: Significant Reduction as Compared to Placebo	Empagliflozin (EMPA-REG) [13]	Canagliflozin (CANVAS trial)[7]	Dapagliflozin (DECLARE-TIMI 58)[12]	Canagliflozin (CREDENCE trial)[9]	Dapagliflozin (DAPA-HF)[20]
Reduction in 3P-ACE indicators	Yes	Yes	No difference	Yes	Not presented
Reduction in CV deaths/hospitalizations due to heart failure	Yes	No	Yes	Yes	Yes
Reduction in all-cause mortality	Yes	No difference	No difference	No difference	Yes
Reduction in the incidence of MI or stroke	No difference	No difference	No	No	No

Discussion

This review aims to compare the results of the five trials and enlighten family physicians to consider the use of these drugs in the community by informing them about the CV outcomes of SGLT2i as people with diabetes are at high risk of developing CV complications despite having reasonable glycemic control. Several systematic reviews and meta-analyses have also reported favorable effects of SGLT2i in reducing fasting blood sugar, HbA1c, blood pressure, lipid profile, and body weight [21-23]. It is, therefore, suggested that SGLT2i drugs can be used as a monotherapy rather than in combination with other anti-diabetic agents [24].

The SGLT2i have shown a promising effect in the prevention of CV events and mortality in patients with pre-diagnosed CVDs. The findings from the first trial EMPA-REG have bought a revolutionary change in the treatment regimens by showing cardioprotective effect by reducing CV death and reduction in hospitalization among heart failure patients [13]. Moreover, the study also reported a reduction in deaths from any cause compared to placebo [13].

The CANVAS trial showed that type 2 diabetics with CVD had a lower risk of death from CV causes [7]. The reduction in 3P-MACE for a composite score came out lower than the EMPA-REG trial, though the participants in both the trials received statins or lipid-lowering medication, antiplatelet, and renin-angiotensin-aldosterone inhibitors (RAASi) [7]. A meta-analysis of six RCTs assessed the efficacy of canagliflozin along with metformin monotherapy and was found that administration of canagliflozin reduces HbA1c [19], but it was found to be associated with an increased risk of below-knee amputation as compared to the placebo. Thus, it is imperative to assess the patients thoroughly before giving the drug [25].

The DECLARE-TIMI 58 trial showed a reduction in CV deaths and hospitalizations for heart failure in diabetics independent of their ASCVD status [26]. Most of the patients didn’t have a history of heart failure and therefore the prevention of new heart failure was also significant. There was no significant reduction in 3P-MACE indicators in the intervention arm. However, dapagliflozin-treated participants did show a protective effect in the renal outcomes as well [12].

In the DAPA-HF trial, dapagliflozin SGLT2i did not individually report on 3P-MACE, but trial results showed a reduction in CV mortality and hospitalizations due to heart failure in all patients independent of whether they had diabetes or not. The trial data did have more favorable outcomes in NYHA (New York Heart Association) class II compared to class III or IV [20]. Also, in the trial, patients were taking ACE /ARB, but the class effect of these drugs did not affect the outcomes in post-hoc analysis. This trial demonstrated that SGLT2i is beneficial in diabetics as well as in non-diabetics [20].

The CREDENCE trial demonstrated the CV benefits of SGLT2i in diabetics with chronic kidney disease. The primary endpoint of the trial consisted of renal events and highlighted a significant reduction in CV deaths and hospitalizations for heart failure patients among participants with no CV risk factors [9]. These results are consistent with the results from the DECLARE-TIMI 58 trial [12]. The trial proved that SGLT2i has other effects independent of their glucose-lowering effect. Hence, it can be perceived that all SGLT2i offer protection against CV events by reducing both mortality and morbidity, especially among heart failure cases.

A meta-analysis conducted by Lo et al. reported that the pooled analysis of the four trials on SGLT2i found an overall 7% and 11% reduction in RR in MACE outcomes and CV death alone, respectively. All-cause mortality was also significantly lower, with an RR of 0.9 (0.84-0.97) in their analysis [27]. Another meta-analysis including three trials by Zelniker et al. found an overall 11% reduction in RR among patients with CV risk factors. Patients with established CVD had a 14% RR, and the risk of CVD or hospitalization for heart failure was reduced by 23% among patients with or without a past history of CVD. However, no significant clinical benefit was seen in patients with multiple risk factors for ASCVD [8]. Moreover, Zou et al. in their meta-analysis of three significant trials showed that SGLT2i treatment was associated with a reduction major adverse CV events (OR = 0.86; 95% CI: 0.80-0.93), myocardial infarction (OR = 0.86; 95% CI: 0.79-0.94), CV mortality (OR = 0.74; 95% CI: 0.67-0.81), and all-cause mortality (OR = 0.85, 95% CI: 0.79-0.92) [28].

Giugliano et al. conducted a meta-analysis on 12 trials involving 120,765 patients and reported that SGLT2i was associated with a 31% reduction in heart failure risk, suggesting a notable decrease [29]. Toyama et al. demonstrated that SGLT2i reduced the risk of CV death, non-fatal myocardial infarction or non-fatal stroke (RR: 0.81; 95% CI: 0.70-0.94), and heart failure (RR: 0.61; 95% CI: 0.48-0.78), without a clear effect on all-cause mortality (HR: 0.86; 95% CI: 0.73-1.01). The results are gathered from 27 studies with around 7,363 participants [30].

All these trials and analyses suggest that SGLT2i have CV benefits independent of their glucose-lowering action but their mechanism of action is unclear [31]. However, the possibility of a reduction in blood pressure, glomerular filtration, albuminuria, and volume of filtrate are all likely to contribute, but further studies are needed to explain the SGLT2i mode of action.

The trials included in this review were large randomized trials conducted in strictly controlled conditions with high rates of drug adherence and close monitoring of adverse events. The follow-up periods were different among all trials, for example, DECLARE-TIMI 58 was conducted for 4.2 years, whereas the median follow-up for the CANVAS trial was 2.4 years. There is a possibility that more prolonged drug administration can influence and produce different study outcomes than given for a smaller duration.

Current guidance suggests that SGLT2i can be used in patients with a past history of CVD, but from the trial data, it is evident that these drugs can be recommended independent of HbA1c level or the presence of ASCVD and can help prevent hospitalization for heart failure [12]. As discussed, these drugs have cardioprotective action, yet a word of caution is needed, especially for primary healthcare providers, before prescribing them to patients. As each drug has its own effect, it is strongly recommended that health practitioners should assess the patient’s profile and also weigh the CV and renal effects of each of these drugs before prescribing them to patients. The National Institute for Health and Care Excellence (NICE) recommends the use of these drugs as a second-line agent if HbA1c remains poorly controlled with metformin and is above 58 mmol/mol (7.5%) and provided that patients are not tolerating sulfonylurea and are at risk of significant hypoglycemia [32]. The latest European Association for the Study of Diabetes (EASD) and American Diabetes Association (ADA) guidelines recommend the use of SGLT2i in diabetic patients with CVD risk factors, history of heart failure, or chronic kidney disease [33]. These can be prescribed as add-on therapy if HbA1c remains poorly controlled or as monotherapy if metformin is poorly tolerated or contraindicated [33].

Some studies report that SGLT2i has some adverse effects and that these drugs are associated with hypotension, dehydration, urinary tract infections, diabetic ketoacidosis, fragility fractures, and risk of lower-limb amputations [25,34,35]. It is recommended not to prescribe these medications in the elderly, patients on diuretics, patients with peripheral vascular disease and history of osteoporosis, and patients with reduced renal function [25,35]. Hence, the patient's age, fragility score, renal status, and other co-morbidities should be considered before prescribing these drugs.

It is clear from the above evidence that SGLT2i is beneficial in terms of CV outcomes by reducing mortality and morbidity and that the health care professionals should consider the use of these drugs more proactively in diabetic patients after considering relevant points as highlighted above. This review considered five major trials with CV outcomes; however, there are further trials ongoing, which will enlighten us further with 3P-MACE indicators, particularly looking at mortality related to myocardial infarction and stroke. Trials on metformin have shown weak evidence [36,37] of their CV benefit, and a recent commentary by Packer [38] has cited evidence that the cardioprotective effect of SGLT2i can be attenuated when given with metformin at the cellular level. But more evidence is needed to study the interaction between the two drugs. This review should help clinicians make evidence-based decisions when treating patients with these drugs.

## Conclusions

This review highlights that SGLT2i, especially empagliflozin, canagliflozin, and dapagliflozin, have significant benefits in reducing CV mortality, heart failure-related hospitalization, and all-cause mortality in patients with T2DM. We can argue that given favorable outcomes and CV benefits of SGLT2i, these drugs can be considered as first-line agents, and trials are needed to specifically prove this. Furthermore, only the aforementioned drugs have demonstrated positive outcomes and therefore we cannot generalize the same effect for all SGLT2i. Clinicians must weigh the benefits and risks of these medications before prescribing these, keeping in mind the ultimate goal, that is, what is best for their patients.
